# Illustrated versus non-illustrated anatomical test items in anatomy course tests and German Medical Licensing examinations (M1)

**DOI:** 10.3205/zma001172

**Published:** 2018-05-15

**Authors:** Olaf Bahlmann

**Affiliations:** 1 Dr. Senckenbergische Anatomie (Institut III), Frankfurt, Germany

**Keywords:** Examination questions, medical illustration, educational measurement, anatomy, anatomy and histology

## Abstract

Illustrated Multiple-choice questions (iMCQs) form an integral part of written tests in anatomy. In iMCQs, the written question refers to various types of figures, e. g. X-ray images, micrographs of histological sections, or drawings of anatomical structures. Since the inclusion of images in MCQs might affect item performance we compared characteristics of anatomical items tested with iMCQs and non-iMCQs in seven tests of anatomy courses and in two written parts of the first section of the German Medical Licensing Examination (M1).

In summary, we compared 25 iMCQs and 163 non-iMCQs from anatomy courses, and 27 iMCQs and 130 non-iMCQs from the written part of the M1 using a nonparametric test for unpaired samples. As a result, there were no significant differences in difficulty and discrimination levels between iMCQs and non-iMCQs, the same applied to an analysis stratified for MCQ formats.

We conclude that the illustrated item format by itself does not seem to affect item difficulty. The present results are consistent with previous retrospective studies which showed no significant differences of test or item characteristics between iMCQs and non-iMCQs.

## Zusammenfassung

Bebilderte Multiple-Choice- (MC) Fragen sind ein integraler Bestandteil von schriftlichen Prüfungen in der Anatomie. In bebilderten MC-Fragen bezieht sich die schriftliche Frage auf verschiedene Typen von Abbildungen wie Röntgenaufnahmen, Mikrofotografien von histologischen Schnitten oder Zeichnungen von anatomischen Strukturen. Da das Hereinnehmen von Abbildungen in MC-Fragen das Abschneiden der Items beeinflussen kann, verglichen wir die Charakteristika von anatomischen Items getestet mit bebilderten und nicht bebilderten MC-Fragen in sieben Anatomieklausuren und in zwei schriftlichen Teilen des Ersten Abschnitts der Ärztlichen Prüfung (M1).

In dieser Studie verglichen wir 25 bebilderte und 163 nicht bebilderte MC-Fragen aus Anatomieklausuren und 27 bebilderte und 130 nicht bebilderte MC-Fragen aus dem schriftlichen Teil des M1 mit einem nicht parametrischen Test für ungepaarte Stichproben. Als Ergebnis waren keine signifikanten Unterschiede im Schwierigkeits- und Trennschärfeniveau zwischen bebilderten und nicht bebilderten MC-Fragen vorhanden, dasselbe ergab sich in einer nach MC-Frageformaten stratifizierten Analyse.

Wir schließen daraus, dass das bebilderte Itemformat für sich die Itemschwierigkeit nicht zu beeinflussen scheint. Die aktuellen Ergebnisse stimmen mit früheren retrospektiven Studien überein, die keine signifikanten Unterschiede zwischen Test- und Itemcharakteristika zwischen bebilderten und nicht bebilderten MC-Fragen zeigten.

## 1. Introduction

Today, there are diverse resources for anatomy assessment. Before the introduction of the Multiple-choice (MC) format in the seventies, state examinations were viva voce [6]. Unstructured oral examinations lack good reliability. Structured oral examinations (SOE) can show good reliability using an item blueprint and a scoring template [13]. 

There are various formats of test items in written assessment. Currently, the most common is the MC format with four or five answer options. In Single-best answer (SBA) questions, there is only one best (correct) answer. SBA is the most popular MC format. In True/false (T/F) items, all correct answers (more than one) must be marked. Simple T/F items might be acceptable. Multiple T/F items with combinations of answer options, used in medical exams in the past, are no longer recommended [4]. The Extended-matching question (EMQ) includes an option list and at least two item stems, and for each stem, the examinee chooses the single best answer from the list. Multiple-choice examinations show high test reliability [6], [13]. Open questions can be answered by a written essay or keywords (Short-answer question, SAQ). Open questions are more time consuming, compared to MC formats [6]. The Modified essay question (MEQ) is a structured variant of the essay format. In spotter/tag tests, MCQs or SAQs refer to marked (tagged) structures in specimens or images [1], [13]. 

Multiple-choice questions (MCQs) are widely used in medical exams. In addition, many medical textbooks nowadays include some self-assessment MCQs at the end of a chapter. The National Board of Medical Examiners (NBME) and other authors published guidelines for the creation of MCQs [4], [8]. Visual resources in exam questions should be accurate, complete, relevant and unambiguous [5]. Instructions on how to produce visual material for MCQs and common pitfalls in anatomy MCQs are published [1], [14]. 

Illustrated MCQs (iMCQs) form an integral part of anatomy tests. Different MCQ formats, e. g. SBA questions or EMQs, can be combined with illustrations. Various illustrations can be included, from x-ray or histological images to photographs of gross preparation specimens or illustrations of functional systems. 

An item analysis shows the difficulty and discrimination of individual MCQs. The difficulty index is the proportion of participants choosing the correct answer. Item discrimination is the correlation between the item score and the test score (item total correlation). Good MCQs have a high correlation coefficient [7], [11]. 

Previous studies did not find significant differences in item or test characteristics between iMCQs and non-iMCQs [3], [9], [12], [15], except for a study on final year students tested with MCQs presenting a clinical problem. In this study on problem-based radiology questions, illustrated items requiring image interpretation were more difficult compared to questions testing recall of knowledge [10]. 

However, the integration of illustrations in MCQs might affect item difficulty and overall test difficulty. Therefore, the aim of the present study was to assess characteristics of illustrated and non-illustrated anatomical items from seven anatomy course tests and two written parts of the first section of the German Medical Licensing Examination (M1) in autumn 2015 and 2016. 

## 2. Methods

### 2.1. Multiple-choice questions 

MCQs from seven consecutive anatomy course tests from winter 2014 to summer 2016 provided the basis for this study. First and second year medical and dentistry students participated in the tests. A test with 30 MCQs was written at the end of course one (musculoskeletal system), course two (internal organs), course three (head and neck and neuroanatomy) and the anatomy seminar for medical students. Between 592 and 364 students participated in the anatomy course tests. Medical students of the Goethe-University Frankfurt wrote M1 examinations with 80 anatomy questions each, in autumn 2015 and 2016 with 393 and 330 participants. Anatomy course tests included between 3 and 7 and the written parts of the M1 12 and 15 illustrated anatomical items. Exam papers were evaluated with EvaExam software (Electric paper, Lüneburg, Germany). 

MCQs classified as doublets and iMCQs with identical illustrations were excluded from the study. Microsoft Excel was used to calculate item difficulty and discrimination from the raw data. The difficulty index was determined as the mean item score. Item discrimination was calculated as the Pearson product-moment correlation coefficient of the individual item score and the sum score of the remaining items (corrected item discrimination). 

Item analyses of M1 questions were produced and are under copyright by the Institute for Medical and Pharmaceutical Exam Questions (IMPP, Mainz, Germany). 

#### 2.2. Statistical analysis 

Data were inspected and tested for normal distribution (Q-Q plot, Shapiro-Wilkinson test). The Kolmogorov-Smirnov test for unpaired samples was used to compare groups of MCQs. Statistical analysis was performed with GraphPad Prism version 7.00 for Windows (GraphPad Software, La Jolla, California, USA). Data were plotted with median and range. A comparison of iMCQs and non-iMCQs stratified for MCQ formats was performed with the stratified van-Elteren U-test (Bias, Version 11.02, epsilon Verlag, 2016).

## 3. Results

**Figure 1:**
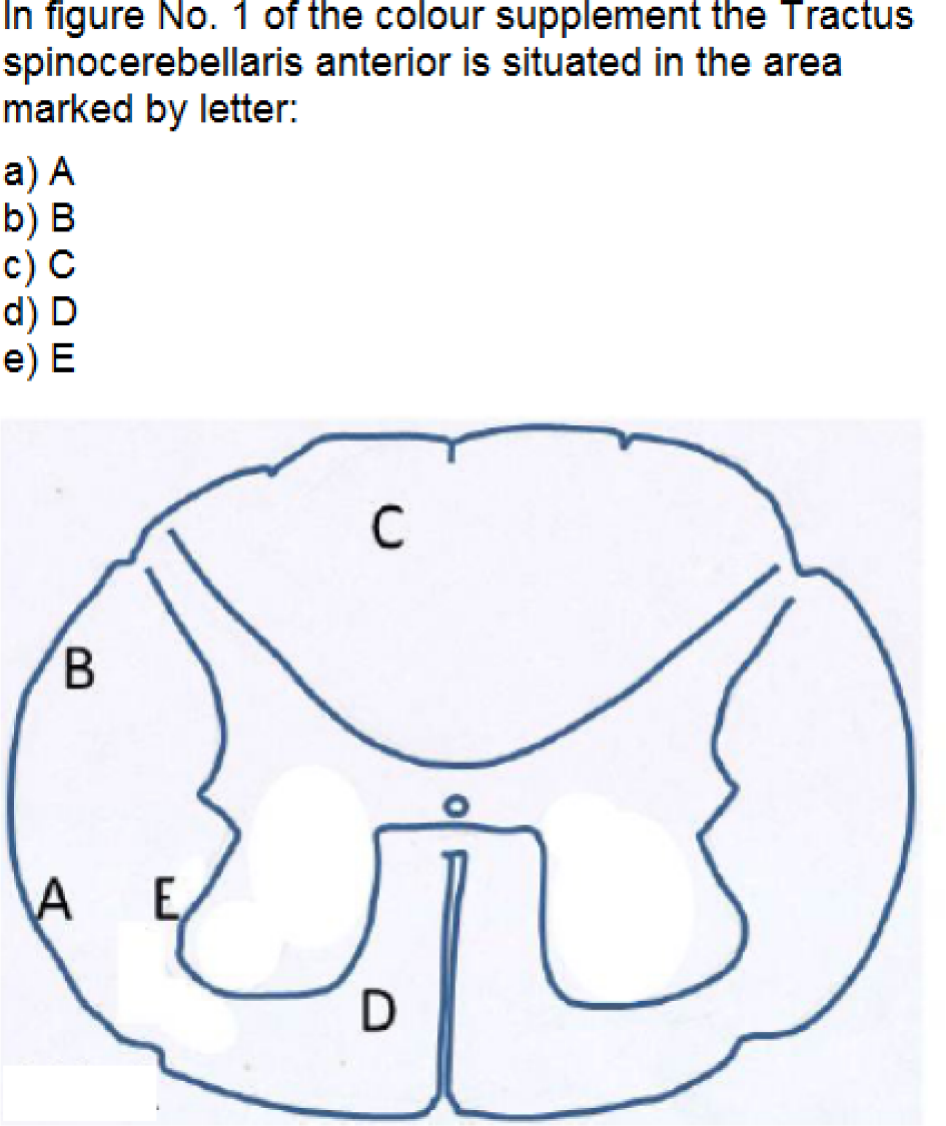
Example of an iMCQ (translation of the original question). The illustrated item format included 13 histological and 5 radiological images, 4 anatomical illustrations, 2 surface anatomy and one brain section image.

From anatomy course tests, 25 iMCQs and 163 non-iMCQs were included in this study. IMCQs consisted of 13 histological and 5 radiological images (conventional x-ray or CT), 4 anatomical illustrations, 2 surface anatomy pictures and 1 image of a gross brain section (see Figure 1 (Fig. 1), translation of the original question). MCQs followed the A-type format (one best answer and four distractors). 

Anatomy questions from two M1 examinations with 27 iMCQs and 130 non-iMCQs were also included in this study. 16 images of histological sections, 8 surface anatomy pictures, 1 image of a dissection specimen, 1 image of a body cross section and 1 anatomical illustration were used in iMCQs. 

In addition, we stratified items according to MCQ formats. The stratified analysis was run on items with a question in the stem and (short) answer options, positively (Group A) or negatively worded (Group B), and MCQs with statements as answer options, positively (Group C) or negatively worded (Group D). Other formats (sentence completion or matching items) were excluded from the analysis. 

**Figure 2:**
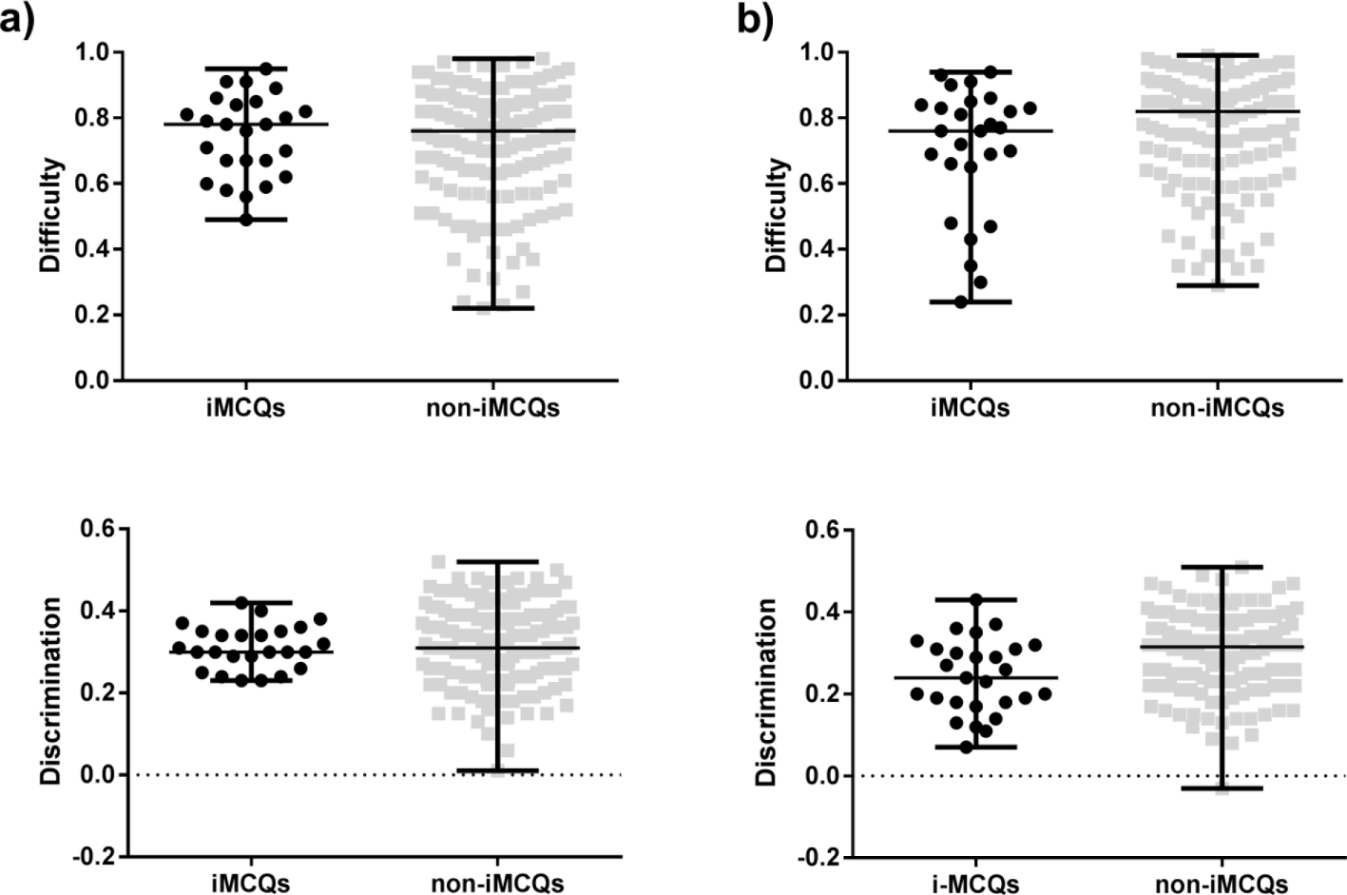
Difficulty and discrimination of iMCQs versus non-iMCQs in anatomy course tests (a) and M1 anatomical items (b). Data shown with median and range. As a result, iMCQs and non-iMCQs were not significantly different in difficulty and discrimination in anatomy course tests and in written parts of the M1.

Median difficulty of iMCQs and non-iMCQs was 0.78 vs 0.76 for anatomy course tests and 0.76 vs 0.82 for the written parts of the M1. The discrimination coefficient was 0.3 vs 0.31 and 0.24 vs 0.315, respectively. As a result, iMCQs and non-iMCQs showed no significant differences in difficulty and discrimination for MCQs of anatomy course tests and written parts of the M1 (p>0.05) (see Figure 2 (Fig. 2)), the same applied to the stratified analysis.

## 4. Discussion

Visual resources are widely used in anatomy teaching and performance assessment. Each anatomy course test includes some iMCQs, and they are part of the written part of the first section of the Medical Licensing Examination (M1). Thus, in the present study, we were interested in the performance of this item format. Therefore, we compared iMCQs and non-iMCQs in anatomy course tests and the written part of the M1. We found that iMCQs and non-iMCQs did not differ significantly in difficulty and discrimination. The fact that iMCQs and non-iMCQs are based on alternative sources of information, i.e. images and text, does not seem to affect item characteristics. 

IMCQs have been assessed previously. Hunt compared two sets of problem-based MCQs in radiology. One set included an image, the other a description of the image, e. g. a radiologist’s report. Final year students wrote the sets in two parallel exams. As a result, the set of items with visual content was significantly more difficult. In Hunt‘s view the results “are consistent with the belief that questions calling for interpretation of data or problem-solving require a higher level of performance or additional skill to that required for questions which supply written descriptions of that data” ([9], p. 420). 

In a study on Part 1 FRACS (Fellowship of the Royal Australasian College of Surgeons) exam questions, the authors compared 77 triplets of MCQs in anatomy and pathology. The MCQs presented four answer options. The triplets consisted of a visual and a verbal question of the same content and an additional verbal one of similar content. There were no significant differences in item difficulty and discrimination. The authors argued that their study was limited by a small sample size and that a lower competence in written English of non-native speaker candidates in the FRACS exams might have influenced the results [3]. 

Vorstenborsch et al. compared 39 EMQs with either an answer list or a labelled anatomical illustration in the item stem. Two test versions were constructed and half of the students wrote each test. Students volunteered for this informal exam, which was similar to the circumstances of an official exam. Using a label, some questions were more and some less difficult, compared to the non-labelled version. Contrary to our study, the authors used extended-matching items instead of MCQs and created closely matched items (labeled image vs answer list). Finally, they were able to compare overall difficulty and reliability of separate test versions. Apart from variable individual effects, the authors did not find overall differences between test versions [15]. 

Holland et al. reviewed histology exams from three consecutive years with 95 iMCQs and 100 non-iMCQs, and found no significant differences in item difficulty or discrimination [9]. In the present study, we included 25 items of all anatomical subjects including 13 histology questions. 

Similarly, in a retrospective analysis on text-only and items with reference images in anatomy examinations, there were no significant differences in difficulty or discrimination between item formats. In this study, the illustration was an addition to the item and did not replace written content, thus images “were considered not to be critical to answering the item” [[12], page 3]. Concerning study design, the studies by Hunt and Vorstenbosch were trial or informal examinations respectively. Students were allocated at random to test groups, and students were not informed about the nature of the examination. Though it was an informal test, test conditions were comparable to an official exam [10]. Each student answered items in both formats [10], [15]. 

The studies by Buzzard and Hunt included radiological items, which went beyond recall of knowledge and asked for thinking in a clinical context (see item examples) [2], [10]. Hunt categorized items according to clinical setting, supplementary data, interpretation, diagnosis and treatment presented in the question stem and options. In all subgroups, items were more difficult in the illustrated format [10]. In the present study, most of the MCQs cover basic anatomical knowledge on a lower cognitive level. 

Hunt showed the increase and decrease of difficulty and discrimination of items created in pairs. 43 out of 70 item pairs increased in difficulty [10]. In the present study, we compared formats of independent items without a pairwise allocation. 

**Figure 3:**
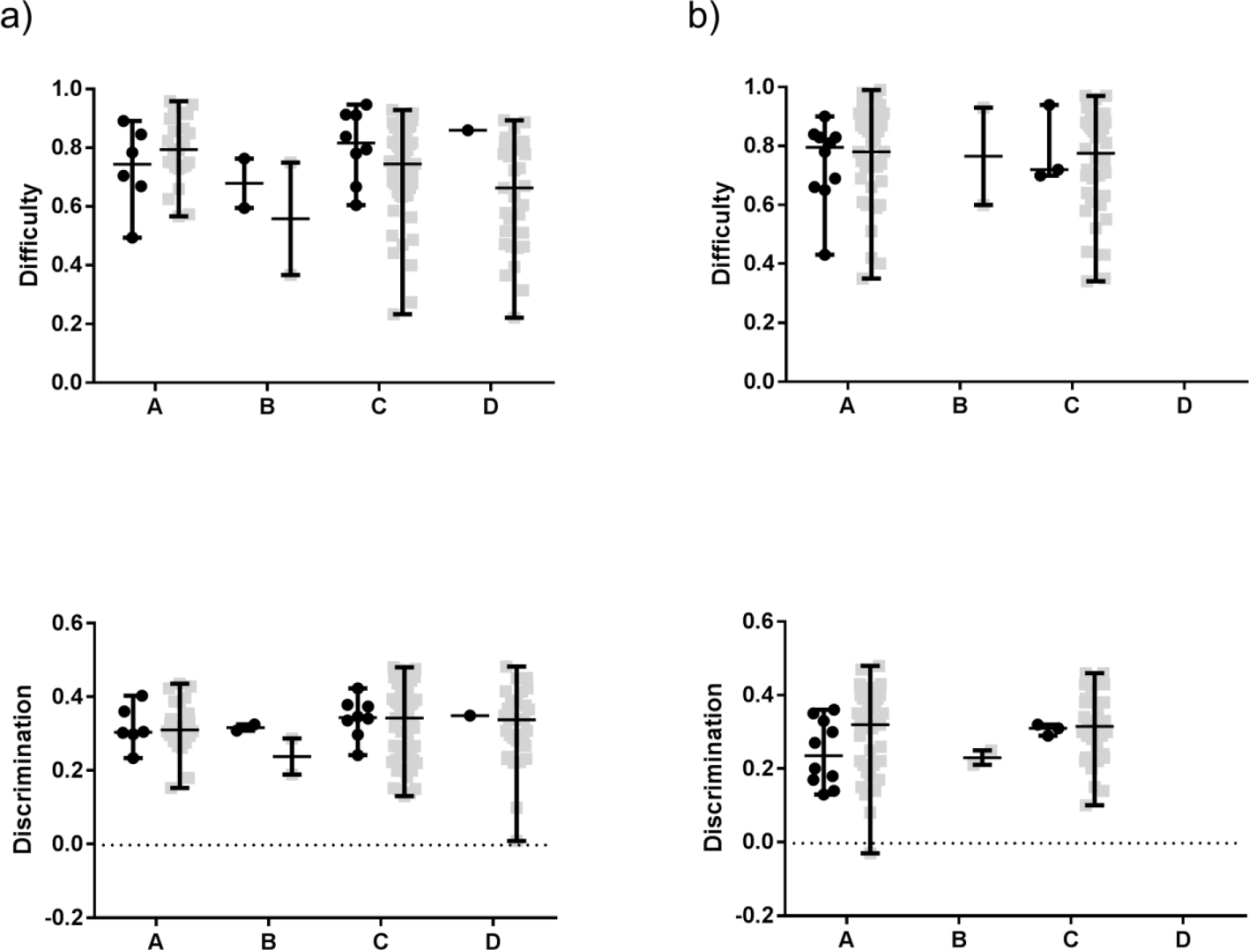
Difficulty and discrimination of iMCQs (●) versus non-iMCQs (□) in anatomy course tests (a) and written parts of the M1 (b), stratified for MCQ formats. Here, MCQs in the (short) answer option format, positively (A) and negatively worded (B), and MCQs in the statement option format, positively (C) and negatively worded (D), are shown, with median and range. As a result, there were no significant differences between iMCQs and non-iMCQs.

In addition, we stratified for MCQ formats (wording and structure of item stems and options) (see Figure 3 (Fig. 3)). However, the integration of illustrations in MCQs had no significant effect on item difficulty and discrimination. 

## 5. Conclusion

In conclusion, iMCQs can be used whenever appropriate. IMCQs can motivate students who are good in visual knowledge and thinking and can be written for lower and higher cognitive levels of exam questions. IMCQs are used to reflect teaching subjects and provide feedback about the effectivity of teaching. Thereby, the introduction of additional visual teaching material can be evaluated by corresponding iMCQs. Using iMCQs, the images must be of sufficient quality and size and accurately labeled. According to constructive alignment, a test blueprint helps choosing iMCQs for the exam. Different kinds of illustrations (histological images, x-rays) will reflect the diversity of visual input in medicine. Checking the quality of iMCQs will also improve students’ learning from trial exam questions. Finally, the results of this study might reassure question writers to use iMCQs.

## Acknowledgements

The author would like to acknowledge Professor Eva Herrmann for statistical advice, Professor Jörg Stehle and Professor Frank Nürnberger for helpful comments and the latter for the iMCQ example. 

## Competing interests

The author declares that he has no competing interests.
